# Autoimmune retinopathy: findings and limitations from optical coherence tomography angiography

**DOI:** 10.1186/s40942-020-00267-4

**Published:** 2020-12-03

**Authors:** Joseph Raevis, Tyler Etheridge, Spencer Cleland, Mihai Mititelu

**Affiliations:** Department of Ophthalmology and Visual Sciences, University of Wisconsin School of Medicine and Public Health, 206 University Ave 2870, Madison, WI USA

**Keywords:** Autoimmune retinopathy, Cancer associated retinopathy, Melanoma associated retinopathy, Optical coherence tomography angiography, OCTA, vessel density, retinal thickness, foveal avascular zone

## Abstract

**Background and objective:**

To report novel findings and limitations from optical coherence tomography angiography (OCTA) in the evaluation of autoimmune retinopathy (AIR).

**Study design:**

We retrospectively reviewed features of five patients diagnosed with AIR and five controls. OCTA scans were obtained and manually segmented to provide accurate measurements of foveal avascular zone (FAZ), vessel density, and retinal thickness at different levels.

**Results:**

The total retina and superficial vessel density throughout the whole scan were similar between AIR and controls (*p* = 0.14 and *p* = 0.11), whereas deep vessel density was decreased in AIR compared controls (*p* = 0.02). Decreased vessel density was most pronounced in the parafoveal and perifoveal areas (*p* = 0.01 and *p* = 0.01). AIR patients also had reduction of total retinal thickness in the perifoveal zone (p = 0.03), corresponding to outer retinal thinning (*p* = 0.001).

**Conclusion:**

This small series shows that AIR patients have reduced deep vessel density, particularly in the parafoveal and perifoveal regions and a decrease in macular thickness. These findings show correlation with the classic “flying saucer” sign seen on OCT.

## Background

Autoimmune retinopathies (AIR) are a heterogenous and often underdiagnosed group of degenerative retinal diseases causing vision loss in the absence of posterior pole pathology such as inherited-retinal disease or overt inflammation. AIR is classified as non-paraneoplastic AIR (npAIR) and paraneoplastic (pAIR) [1], with the latter being further classified as cancer associated retinopathy (CAR) and melanoma associated retinopathy (MAR) [2].

Non-paraneoplastic autoimmune retinopathy is the most common subtype and is frequently associated with an underlying autoimmune etiology such as thyroid or connective tissue diseases. Acute zonal occult outer retinopathy is a considered subtype of npAIR and can show a trizonal pattern of retinal and retinal pigment epithelium (RPE) degeneration [3].

CAR is the most common paraneoplastic retinopathy and is associated with small cell lung cancer but can also be seen with genitourinary cancers [4]. Visual symptoms precede the diagnosis of a systemic malignancy in approximately 50% of cases, highlighting the need for a systemic workup whenever there is suspicion for CAR [1, 3, 5]. MAR is frequently observed in patients with a prior diagnosis of cutaneous or uveal melanoma [3].

Patients typically present with bilateral, progressive decreased best corrected visual acuity (BCVA), photopsia, visual field constriction, and scotomas [2]. Fundus findings are commonly normal [6], but vascular attenuation, RPE atrophy, optic disc pallor may be present to varying degrees [7].

Antiretinal antibodies (ARA) are believed to be an important component of the pathophysiology of AIR. Still, the detection of isolated ARAs is not pathognomonic for AIR, as these antibodies can be seen in otherwise healthy individuals [3]. Moreover, there have been reports of AIR cases without any positive antibodies detected [8]. The most commonly identified antibodies include anti-recoverin (23 kDa), which is a photoreceptor-specific calcium binding protein that may induce retinal apoptosis, anti-alpha-enolase (46 kDa), and anti-carbonic anhydrase II (30 kDa) [4, 9].

Optical coherence tomography angiography (OCTA) is a non-invasive and relatively new technology that uses the OCT platform to improve our understanding of retinal microvascular anatomy and blood flow [10]. Specifically, OCTA has allowed visualization of the deep capillary plexus, which was previously not possible only with fluorescein angiography [11]. Very little has been written about the OCTA findings in AIR, with knowledge of the OCTA changes in this condition being limited to two published case reports [12, 13]. The aim of this study is to report for the first time the OCTA findings of a small series of patients with AIR, to compare these findings to normal age matched controls and to discuss the challenges and limitations associated with the use of this technology when imaging AIR.

## Methods

### AIR diagnosis

A retrospective medical record review was performed to identify patients with the diagnosis of AIR, pAIR, npAIR and age matched controls seen at the University of Wisconsin-Madison, Department of Ophthalmology and Visual Sciences, Eye Clinics between January 2012 and March 2020. The study was approved by the institutional review board at the University of Wisconsin-Madison, adhered to the tenets of the Declaration of Helsinki and complied with the Health Insurance Portability and Accountability Act.

The diagnosis of AIR was made based on a combination of medical and ocular history, ophthalmic examination, structural and functional multimodal testing and antibody panel (Table 1). We followed the criteria set by Ferreyra et al. that categorize evidence into “strong,” (diffuse retinal atrophy, a negative electroretinogram (ERG), a diagnosis of prior cancer (for CAR) and a history of autoimmune disease in 50% of patient’s immediate family) “supportive,” or “helpful” (abnormal ERG findings with typical symptoms such as quick onset photopsias with normal vision prior to onset, rapid progression by history of vision or visual fields, and multiple antiretinal antibodies bands on western blot) [14]. Healthy aged match controls were selected randomly from a database of patients without an ocular or systemic disease evaluated using the same testing modalities. Antibody testing was completed through the Ocular Immunology Laboratory at Oregon Health and Science University (Portland, OR).

**Table 1:** Clinical and demographic information

Patient	Age	Gender	Eye	BCVA	Systemic diseases	Treatment	Antibodies	Additional AIR diagnostic criteria
Control 1	85	Female	OD	20/25	None	None	Not done	
Control 2	64	Male	OD	20/20	None	None	Not done	
Control 3	74	Male	OD	20/25	None	None	Not done	
Control 4	69	Female	OD	20/25	None	None	Not done	
Control 5	49	Female	OD	20/20	None	None	Not done	
AIR 1	89	Female	OS	CF at 3’	None	Azathioprine	Not done	Diffuse retinal atrophy, flat ffERG, normal vision prior to onset, rapid progression of visual field loss, subtle “flying saucer sign”
AIR 2	49	Female	OS	20/20	Multiple sclerosis	Mycophenolate azathioprine	22, 30, 42, 44, 62, 72, 136 kDa	Diffuse peripheral retinal atrophy, rapid progression of visual field loss, photopsias, classic OCT “flying saucer sign”
AIR 3	76	Female	OD	8′/200	Hashimoto’s thyroiditis	Mycophenolate	30, 45, 46 kDa	Decreased amplitudes on ffERG and mfERG, rapid progression of visual field loss, photopsias, subtle “flying saucer sign”
CAR 4	79	Female	OD	20/40	PMR, Hypothyroid, Breast Cancer	Observation	30, 46 kDa	Flat ffERG, rapid progression of visual field loss
AIR 5	75	Female	OS	LP	Hypothyroidism, RA	Observation	30, 33, 60, 70 kDa	Reduced → flat ffERG, photopsias, normal vision prior to onset, rapid progression of visual field loss until LP

*AIR* Autoimmune Retinopathy, *CAR* Cancer Associated Retinopathy, *BCVA* Best Corrected Visual Acuity, *CF* Count Fingers, *LP* Light Perception, OD Right eye, *OS* Left eye, *PMR* Polymyalgia Rheumatica, *RA* Rheumatoid Arthritis, *ffERG* Full Field Electroretinogram, *mfERG* multifocal electroretinogram. Age corresponds to age of image acquisition

### OCTA methodology

OCTA scans were obtained using the commercially available system RTVue Avanti OCTA (Optovue Inc, Fremont, California, USA). The Optovue scans comprised 304 A-scans by 304 B-scans and 400 A-scans by 400 B-scans for high-definition 6 × 6 mm scans for each control and AIR patients. Scans were evaluated to ensure high-resolution images and capture of the full Early Treatment Diabetic Retinopathy Study (ETDRS) grid, which permits assessment of the perifovea. OCTA scans were reviewed in the proprietary software (Optovue review software, version 2018.0.05). Quality index was documented from Optovue scans using signal quality (SQ 1–10) and signal strength index (SSI 1–100). Scans with a SQ less than 5 and/or SSI less than 50 were excluded [15].

**Fig. 1:**
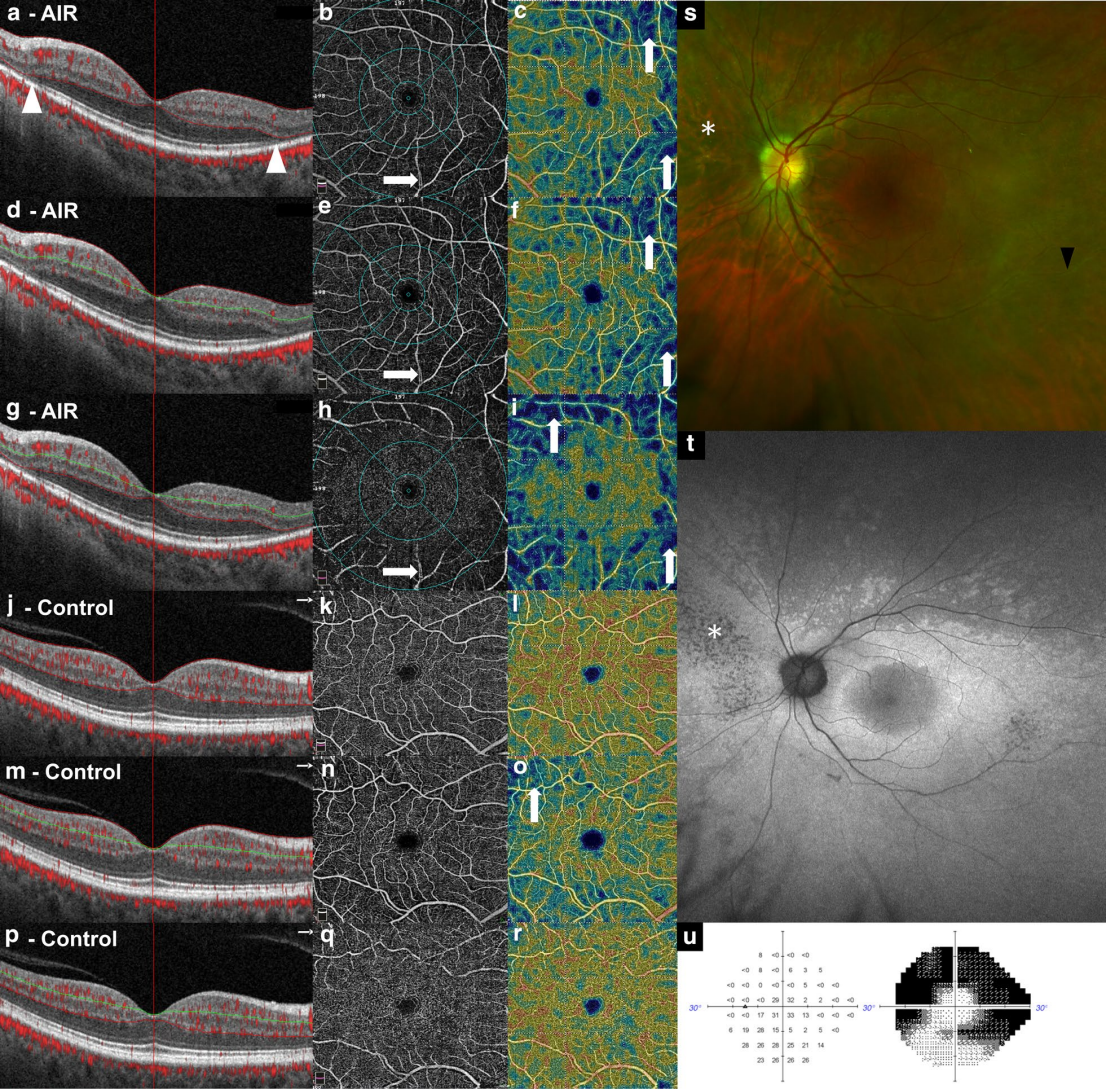
Multimodal images from a patient’s eye with autoimmune retinopathy **a**–**i**, **s**–**u** and a normal control (**j**–**r**). Foveal centered B-scans **a**, **d**, **g**, **j**, **m**, **p** with vessel identification, angiogram **b**, **e**, **h**, **k**, **n**, **q** with vascular markings and vascular density map **c**, **f**, **i**, **l**, **o**, **r** are shown. **a**, **j** Retina slab showing superficial and deep capillary plexuses. **d**, **m** Superficial slab showing superficial capillary plexus. **g**, **p** Deep slab showing deep capillary plexus. The OCT shows loss the of external limiting membrane, ellipsoid zone and photoreceptors in her periphery (white arrowheads). Note the presence of tilt artifact in the foveal centered B-scan (vertical arrows) and vessel doubling due to eye movement (horizontal arrows) in the angiogram with vascular markings. The color fundus wide-field photograph **s** reveals attenuated vessels (black arrowhead) and diffuse retinal atrophy (asterisk), both of which are also noted on the fundus auto fluorescence (**t**). A 24-2 Humphrey visual field shows peripheral, concentric, field loss with central and inferior sparing (**u**)

The proprietary software automatically segmented the retinal vasculature into two slabs (superficial capillary plexus and deep capillary plexus), followed by manual segmentation and grading by two post-graduate imaging fellows. The boundaries of the slabs included the innermost aspect of the internal limiting membrane to bottom on the outer plexiform layer (total retina), innermost aspect of the internal limiting membrane to the outermost layer of the inner plexiform layer (inner retina), and outermost layer of inner plexiform layer to the bottom of the outer plexiform layer (outer retina) (Fig. 1). OCTA scans from the worse affected eye of AIR patients (n = 5) were compared to the right eye of controls (n = 5) for analysis.

Using the nine sectors of the ETDRS grid the mean vessel density was automatically calculated for each slab from the fovea (1 mm diameter), parafovea (3 mm diameter), and perifovea (6 mm diameter). The foveal avascular zone (FAZ, mm^2^) was automatically delineated and calculated within the total retina slab. Decentration of the OCT grid was manually repositioned when required.

Retinal thickness (μm) was automatically calculated for the total retina (internal limiting membrane to retinal pigment epithelium), inner retina (internal limiting membrane to inner plexiform layer), and outer retina (inner plexiform layer to the retinal pigment epithelium).

In addition to the SQ and SSI, OCTA images were reviewed for the presence of a range of previously reported OCTA artifact, including ETDRS grid centration, eye movement, refractive shift, defocus, shadow, z-offset, tilt, projection, and the presence of blink lines [16]. The severity of each artifact was graded as mild, moderate, and severe, if present.

### Statistical analysis

Continuous variables were expressed as means and percentages. Exploratory analysis revealed a normal distribution for the FAZ, vessel density, and retinal thickness measurements. A student’s t-test was performed to compare mean measurements between AIR and controls. The mean vessel density within the whole image, fovea, parafovea, and perifovea were compared instead of individual sectors to limit the problem of multiple comparisons. Similarly, the mean retinal thickness within the fovea, parafovea, and perifovea were compared instead of individual sectors. Statistical analysis was performed using Microsoft Excel (Microsoft Corp).

## Results

### Patient characteristics

The mean ± standard deviation age was 73.7 ± 15.0 years for AIR patients compared to 68.0 ± 13.2 years for controls (*p* = 0.54). Each AIR patient had either one or two of the strong evidence factors and between two and four of the supportive criteria set forth by Ferreyra et al. [14] Four of the five AIR patients had antibody testing done, and each of the four patients that were tested had multiple AIR associated antibodies (Table 1).

### Scan quality and artifact

Of the 10 scans available, all met SQ and SSI inclusion criteria and were included in the analysis. The mean SQ and SSI did not differ between AIR and controls (6.8 ± 0.9 vs 7.8 ± 1.3, *p* = 0.19; 57 ± 2.9 vs 66 ± 11.2, *p* = 0.14) (Table 2). Every OCTA image, for both AIR and control groups, contained several artifacts, including decentration of the ETDRS grid, eye movement, refractive shift, defocus, shadowing, z-offset, tilt, projection, and blink lines (Additional file 1: Table S1). For both AIR and controls the artifacts were mild to moderate in severity, with the exception of the OCTA scan for patient “AIR 5”, which contained one severe artifact (decentration), related to the patient’s inability to fixate due to light perception vision.

**Table 2:** Comparison of foveal avascular zone and vessel density between autoimmune retinopathy and controls

	Controls	AIR	*p* value
Eyes, no	5	5	
Scan quality (1–10), mean (SD)	7.8 (± 1.3)	6.8 (± 0.8)	0.19
Signal strength index (1–100), mean (SD)	66.0 (± 11.2)	57.0 (± 2.9)	0.14
FAZ (mm^2^), mean (SD)	0.194 (± 0.040)	0.290 (± 0.154)	0.21
Vessel density (%), mean (SD)			
Total retina			
Whole image	52.5 (± 5.0)	46.3 (± 6.6)	0.14
Fovea	39.4 (± 6.5)	32.4 (± 7.4)	0.15
Parafovea	56.4 (± 5.4)	49.6 (± 7.1)	0.12
Perifovea	52.6 (± 5.4)	44.8 (± 6.0)	0.08
Inner retina			
Whole image	48.5 (± 3.1)	43.9 (± 4.9)	0.11
Fovea	25.3 (± 5.4)	25.0 (± 6.8)	0.94
Parafovea	51.7 (± 4.4)	46.9 (± 5.9)	0.18
Perifovea	48.4 (± 3.1)	43.3 (± 4.5)	0.08
Outer retina			
Whole image	49.5 (± 6.4)	39.4 (± 4.4)	*0.02*
Fovea	43.6 (± 6.0)	36.0 (± 7.3)	0.11
Parafovea	53.7 (± 4.4)	44.6 (± 4.2)	*0.01*
Perifovea	50.4 (± 7.4)	37.7 (± 1.5)	*0.01*

Vessel density is represented as a percentage with standard deviation (SD)

Italic values indicate statistical significance at *p* < 0.05

### FAZ

**Fig. 2:**
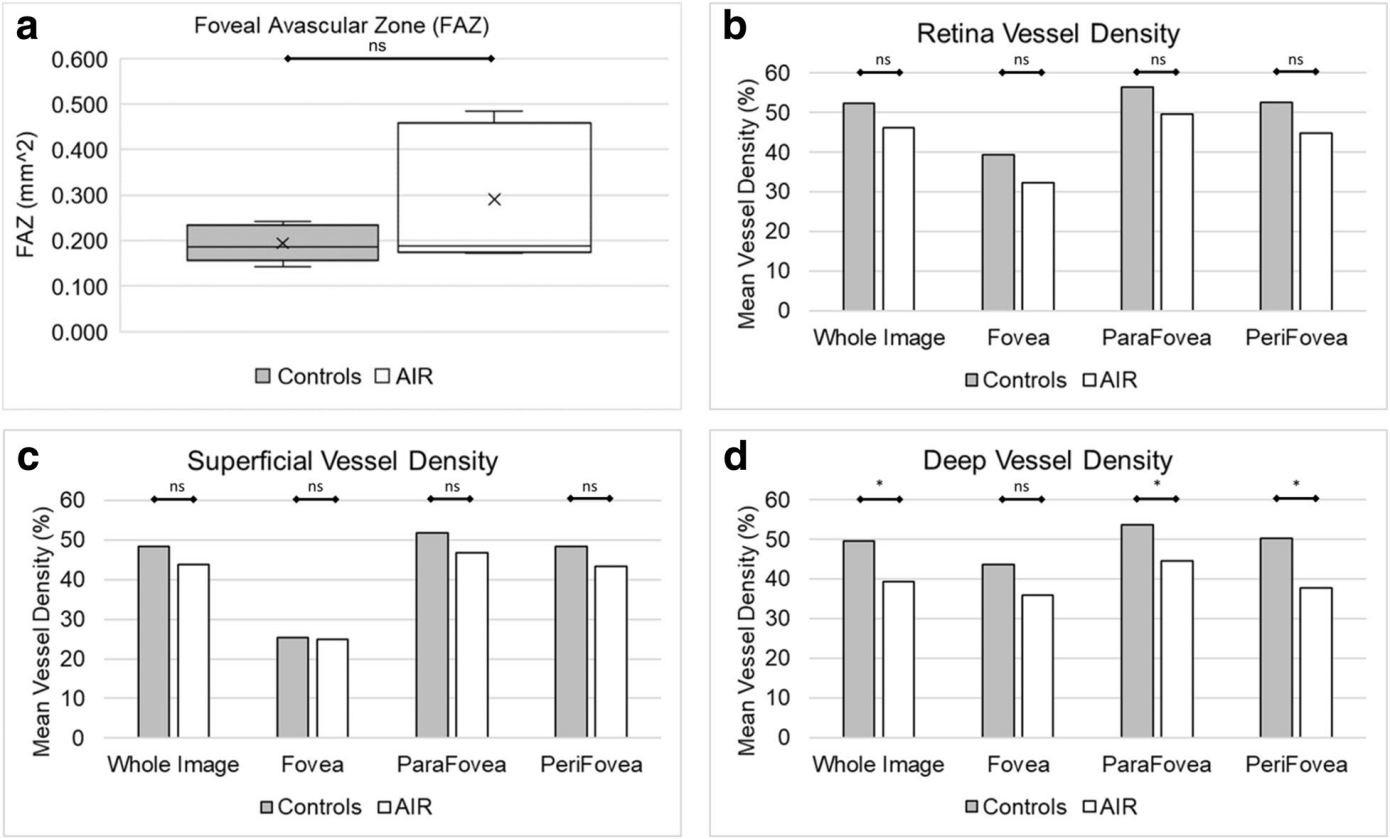
Comparison of **a** foveal avascular zone, **b** mean vessel density for the retina slab, **c** mean vessel density for the superficial slab, and **d** mean vessel density for the deep slab between autoimmune retinopathy (AIR) and age matched controls. *ns* not significant, significant = p < 0.05 (*)

FAZ mean was 0.290 ± 0.154 mm^2^ in AIR compared to 0.194 ± 0.040 mm^2^ in controls (*p* = 0.21) (Table 2 and Fig. 2a).

### Vessel density

Mean vessel density throughout the whole image for the retina slab was 46.3 ± 6.6% in AIR compared to 52.5 ± 5.0% in controls (*p* = 0.14) (Table 2 and Fig. 2b). Mean vessel density throughout the whole image for the superficial capillary plexus was 43.9 ± 4.9% in AIR compared to 48.5 ± 3.1% in controls (*p* = 0.11) (Table 2 and Fig. 2c). Mean vessel density throughout the whole image for the deep capillary plexus was 39.4 ± 4.4% in AIR compared to 49.5 ± 6.4% in controls (*p* = 0.02) (Table 2 and Fig. 2d). The decreased vessel density in the deep capillary plexus in AIR compared to controls was marked in the parafovea (44.6 ± 4.2% vs 53.7 ± 4.4%, *p* = 0.01) and perifovea (37.7 ± 1.5% vs 50.4 ± 7.4%, *p* = 0.01).

### Retinal thickness

Mean total retinal thickness was significantly decreased in the perifovea of AIR patients at 229 ± 19 μm compared with controls of 267 ± 19 μm (*p* = 0.03). No difference was found in total retinal thickness in the fovea or parafovea. There was no difference between AIR and control patients within the inner retina, however a significant thinning was identified within the outer retina in the perifovea region. The outer retinal perifovea of control patients was 175 ± 9 μm compared with 122 ± 5 μm in AIR (*p* = 0.0001). No difference was found in the outer retinal thickness in the fovea or parafoveal regions (Table 3).

**Table 3:** Comparison of retinal thickness between autoimmune retinopathy and controls

	Controls	AIR	*p* value
Thickness (μm), mean (SD)			
Total retina			
Fovea	254 (± 8)	217 (± 51)	0.14
Parafovea	309 (± 10)	259 (± 84)	0.22
Perifovea	267 (± 19)	229 (± 19)	*0.03*
Inner retina			
Fovea	59 (± 4)	62 (± 10)	0.59
Parafovea	103 (± 5)	101 (± 36)	0.92
Perifovea	92 (± 10)	107 (± 7)	0.07
Outer retina			
Fovea	205 (± 6)	165 (± 45)	0.08
Parafovea	204 (± 6)	156 (± 48)	0.06
Perifovea	175 (± 9)	122 (± 15)	*0.001*

Italic values indicate statistical significance at *p* < 0.05

*SD* Standard deviation

Mean total retinal thickness was significantly decreased in the perifovea of AIR patients at 229 ± 19 μm compared with controls of 267 ± 19 μm (*p *= 0.03). No difference was found in total retinal thickness in the fovea or parafovea (*p* = 0.14, *p* = 0.22). There was no difference between AIR and control patients within the inner retina, however a significant thinning was identified within the outer retina in the perifovea region (*p* = 0.0001). No difference was found in the outer retinal thickness in the fovea or parafoveal regions (Table 3).

## Discussion

In this study we report novel OCTA findings in a cohort of five AIR patients compared to normal healthy age matched controls. To our knowledge, prior OCTA findings in AIR patients have been limited to two case reports. A case of npAIR was reported by Kasongole et al. showing OCTA void areas in the choroidal vasculature [12]. A case of MAR reported by Patel et al. showed perifoveal vessel dropout on the OCTA, both the superficial and deep retinal layers, and perifoveal small vessel dropout but the FAZ was not measured [13]. Both studies were descriptive, did not have a control group and did not obtain objective measurements of the OCTA scans.

OCT findings that have been reported with AIR include the classic “flying saucer” sign with parafoveal attenuation of the outer nuclear layer, external limiting membrane and ellipsoid zone in the presence of subfoveal preservation of these outer retinal elements [3, 16]. Other OCT findings include non-leaking intraretinal cystic spaces, retinal pigment epithelium and choriocapillaris atrophy and decreased macular retinal thickness [13], although up to 18% of AIR cases can have normal SD-OCT findings [3].

Our OCTA series provides novel insights into the characteristics of the “flying saucer” sign. When evaluating vascular density (Table 2), our OCTA analysis revealed decreased vessel density in the outer retina, specifically in the parafoveal and perifoveal regions of the macula. These findings on OCTA correspond to the same locations as the observed retinal thinning on OCT. A similar trend was noted when evaluating retinal thickness on OCTA (Table 3). We confirm the decrease in the total retinal thickness of the macula of patients with AIR and show that the thinning is most pronounced in the outer retina, and specifically in the perifoveal region. This loss of the outer retinal segments is consistent with the mechanism of antiretinal antibodies affecting the photoreceptors and leading to gradual retinal degeneration, a process most notable in the outer retinal layers.

AIR has historically not been regarded as a primarily vascular disease. Instead, the condition is believed to be triggered by molecular mimicry, a phenomenon in which there are similarities in sequence between foreign antigens and self-antigens such that self-antigens can cause an immune response [17]. In paraneoplastic retinopathies such as CAR, molecular mimicry occurs between tumor antigens and retinal proteins, whereas in nPAIR, the mimicry is between retinal proteins and presumed infectious (bacterial or viral) or inflammatory antigens. As such, OCTA findings from our study tend to suggest that the decreased vessel density of the outer retina is a result of the retinal degeneration typically seen with AIR.

There is debate in the literature on whether OCTA can serve as a non-invasive imaging alternative for fluorescein angiography (FA) in retinal vascular conditions such as diabetic retinopathy or venous occlusive disease. In AIR, these two modalities may work synergistically: FA has shown retinal pigment epithelium defects, retinal vascular inflammation and leakage [18], while OCTA may provide a more granular look at the thickness and microvasculature health of individual retinal layers.

AIR is a diagnosis that is poorly understood and requires multiple tests and a high index of suspicion to hone the diagnosis. There are no standard clinical and laboratory guidelines for this entity, which requires better understanding and improved consensus on its diagnosis and management. Multimodal imaging has been helping clinicians to identify specific disease patterns in AIR, and we believe that the addition of the OCTA to the already existing armamentarium will add a new dimension to our understanding of AIR. Specifically, OCTA provides an understanding of tissue thickness and vascular density which we believe represent biomarkers of macular health in AIR and may facilitate the diagnosis and clinical follow-up of patients with this condition.

The retinal degeneration seen with AIR can be progressive, and the goal of treatment is the arrest of the disease progression, the prevention of contralateral eye involvement and in rare cases the improvement in visual acuity and visual field [3]. Further research on the use of OCTA is needed to determine whether changes in vascular density or retinal thickness correlate with response to treatment, similar to the potential correlation between treatment effect of decreasing circulating antibodies and improvement in visual function.

### Limitations

This study is limited by its small size of only five AIR patients, due to the rarity of the disorder. The statistically significant results shown are due to very pronounced differences between the normal eyes and AIR patients.

Another limitation of our study, just like with other AIR studies, is that this represents a diagnosis of exclusion. No definitive test exists to conclusively diagnosis AIR. We included only patients with multiple criteria set forth by Ferreyra et al. using complex medical and ocular histories and rigorous multimodal testing (Table 1) [10].

Due to the severity of the retinal disease seen in the study eyes, time-intensive manual segmentation and grading had to be performed for each OCT slice. We used 6 × 6 scans because of their ability to examine a larger area of the macula and thus identify more peripheral pathology that would not otherwise be caught by 3 × 3 scans.

It has been shown that OCTA images are commonly affected by artifact that may impair quantitative outputs. [19] Every OCTA image from both AIR and controls contained multiple artifacts, but the vast majority of these artifacts were mild to moderate in severity (Additional file 1: Table S1). Eye movement and refractive shift were the most severe artifacts encountered, which can be expected given the long acquisition time of OCTA images (Additional file 1: Table S1). We attempted to mitigate this inherent limitation of OCTA by setting stringent SQ and SSI requirements. Future, more comprehensive studies could look at the role of software dedicated to minimizing artifacts and also at OCTA platforms different than the RTVue Avanti system used in this study in order to provide a comparison in terms of the nature and range of artifacts seen in this retinal pathology.

Finally, our study demonstrates that OCTA artifacts remain prevalent when imaging AIR cases. Since these cases often present with retinal degeneration and decreased visual function, the use of OCTA can be particularly challenging, and clinicians need to recognize the range of artifacts and limitations in order to properly interpret the imaging findings. Correlating the OCTA findings with those obtained from multimodal testing (OCT, ERG, FA, visual fields, etc.) often employed in AIR [3] will likely provide the best imaging tools for better understanding this difficult condition in clinical practice.

## Conclusions

In the current study, AIR patients had decreased vessel density in the deep capillary plexus, particularly in the parafoveal and perifoveal zones. The macular thickness of AIR patients was also decreased in the perifoveal region of the outer retina. These angiographic findings correlate with the classic “flying saucer” sign described on OCT and are consistent with previously hypothesized disease mechanisms where antiretinal autoantibodies target the outer retina and lead to retinal degeneration and vision loss. OCTA analysis in AIR remains challenging due to the artifacts associated with this imaging modality and the extensive retinal degeneration and poor visual function of these cases. Nonetheless, OCTA can provide unique insights into the pathophysiology of AIR and given its rapid and non-invasive nature may become part of the multimodal imaging armamentarium employed in the diagnosis and management of AIR.

## Supplementary information

**Additional file 1:** **Table S1.** OCTA quality of each patient’s scan.

## Data Availability

All data generated or analyzed during this study are included in this published article and its supplementary information files.

## Abbreviations

AIR: Autoimmune retinopathies
npAIR: Non-paraneoplastic AIR
pAIR: Paraneoplastic
CAR: Cancer associated retinopathy
MAR: Melanoma associated retinopathy
RPE: Retinal pigment epithelium
BCVA: Best corrected visual acuity
ARA: Antiretinal antibodies
OCT: Optical coherence tomography
OCTA: Optical coherence tomography angiogram
ERG: Electroretinogram
ETDRS: Early Treatment Diabetic Retinopathy Study
FAZ: Foveal avascular zone
SQ: Signal quality
SSI: Signal strength index
