# Supplementary material for: Autoimmune retinopathy: findings and limitations from optical coherence tomography angiography
Source: Int J Retina Vitreous. 2020 Dec 3;6:64. doi: 10.1186/s40942-020-00267-4 (PMC7713440; doi:10.1186/s40942-020-00267-4)
Supplement: Supplementary file 1 — Additional file 1: Table S1. OCTA quality of each patient’s scan. [file 40942_2020_267_MOESM1_ESM.docx]

**Table S1 (supplemental):** OCTA scan quality

| Subject | Eye | SQ | SSI | Centration | Eye Movement | Refractive Shift | Defocus | Shadow | Z-Offset | Tilt | Projection | Blink Lines |
| --- | --- | --- | --- | --- | --- | --- | --- | --- | --- | --- | --- | --- |
| Control 1 | OS | 6 | 55 | Mild | Mod | Mod | Mod | Mod | Mil | Mild | Present | Mod |
| Control 2 | OS | 7 | 58 | Mild | Mod | Mild | Mild | Mild | Mild | Mild | Present | Mild |
| Control 3 | OD | 8 | 62 | Mod | Mod | Mod | Mod | Mod | Mild | Mild | Present | Mild |
| Control 4 | OD | 6 | 55 | Mild | Mod | Mod | Mild | Mild | Mild | Mild | Present | Mod |
| Control 5 | OS | 7 | 57 | Severe | Mod | Mod | Mild | Mild | Mild | Mild | Present | Mild |
| AIR 1 | OD | 6 | 55 | Mild | Mild | Mild | Mild | Absent | Mild | Mild | Present | Absent |
| AIR 2 | OD | 8 | 59 | Mild | Mild | Mild | Mild | Mod | Mild | Mild | Present | Mild |
| AIR 3 | OD | 9 | 80 | Mild | Mild | Mild | Mild | Mod | Mild | Mild | Present | Mild |
| CAR 1 | OD | 7 | 60 | Mild | Mild | Mod | Mild | Mod | Mild | Mild | Present | Mild |
| AIR 4 | OD | 9 | 76 | Mild | Mild | Mild | Mild | Absent | Mild | Mild | Present | Mild |

**Supplemental Table S1:** OCTA quality of each patient’s scan. Scan Quality (SQ) range 0-10; Signal Strength Index (SSI) range 0-100, Moderate (Mod).
